# Notes on Medical Therapeutics

**Published:** 1886-03

**Authors:** Arthur B. Prowse

**Affiliations:** Assistant Physician, Bristol Royal Infirmary


### NOTES ON MEDICAL THERAPEUTICS. By

ARTHUR B. PROWSE, M.D. Lond., F.R.C.S. Eng.; Assistant Physician, Bristol Royal Infirmary.

INTRA-THORACIC ANEURISM.—Dr. C. M. Richter, in the *Pacific Med. and Surg. Journal and Western Lancet*, March, 1885, records his experience of two cases of the above treated by galvano-puncture. He mentions the number of published cases as 151, of which 40 were treated by the continuous, and 111 by the interrupted, current. In 81 of these, improvement, to a greater or less extent, occurred. Chambers, in the *American Journal of Medical Sciences* (1883), assigns a mortality of 12.9 per cent. to cases treated by galvano-puncture, as compared with 33·1 per cent. where ligation was employed. The author used a 20-cell battery (by Fleming, of Philadelphia), with a galvanometer, "to be sure about the current." The needle—about 3½ inches long, of polished steel, and uninsulated—was attached to the *positive* pole. Anæsthesia was discarded. The needle was inserted to the depth of from ⅔ to 2 inches, in the least resistant part of the tumour. A sponge formed the negative pole, and was placed on the skin "near the tumour." "The current is then gradually increased from the first to the twentieth element inside of from one to two minutes. The current is now further increased by means of the rheostat until the patient complains of severe pain. This strength is maintained for eight minutes, when it is gradually reduced during about one minute." As a rule, the needle is only inserted once during one *seance*. In the first case, a man aged 35, the aneurism had existed about eighteen months; it bulged in the second right intercostal space, and was rapidly increasing. Pot. iod. had been given, without benefit; for one or two weeks the daily dose was one ounce. Between November 12th, 1883, and May 11th, 1884, galvano-puncture was performed six times. The first operation relieved entirely the severe pain in the chest; and eventually the tumour diminished to one-half its former size, and the man was able to enter upon a life of active exertion. In the other patient, aged 69, the aneurism had existed fully six years; it bulged above the sternum, and the physical signs were most urgent. The first galvano-puncture gave so much relief that it was repeated on two occasions. The patient, however, slowly sank, and died, one week after the third operation, from exhaustion. After death the sac was found completely filled with firm laminated clot.

THE FORMATION OF FAT.—Recent German work on the question of the sources of body fat is summarised by F. Gehrig in a paper, "On the Formation of Fat" (*Deutsche med. Wochenschrift*, No. 10, 1885). The sources of the fat of the body are: (1) The fat of the food; (2) the albumen; and (3) the carbo-hydrates. According to Rübner, 100 parts of fat, 211 of albumen, and 232 of carbo-hydrates are of equal value in its formation. Lebedeff finds that milk may contain a fat foreign to that of the body, if such fat be taken as food. Munk has established the fact that the fatty acids are easily emulsionised in a little soap, and that this takes place during digestion. He concludes, also, that much of the neutral fat taken as food is split up into glycerine and fatty acids, and only about one-tenth is absorbed as such. In the thoracic duct, however, very little free fatty acid is found, though plenty of neutral fat. S. Chaniewsky's experiments on geese prove conclusively that fat is formed from carbo-hydrates. He also notices that formation of fat takes place more rapidly in animals previously starved, *i.e.*, whose tissues and blood are poor in albumen.

"THE TREATMENT OF CORPULENCY."—Discussion of the "Kongress für innere Medicin," held at Wiesbaden, April, 1885 (*Beitrage zum Centralblatt für klin. Medicin*, No. 20, 1885).—In 1883, Voit recommended that little fat and carbo-hydrates, but much albumen, should be given.

Ebstein recommends more fat than Voit allows; and, in limiting the amount of food, prefers to lessen the carbo-hydrates rather than the fat. One advantage of doing so is, that fat lessens the feeling of hunger and thirst more than other foods do. He reduces, also, the amount of fluid, and recommends active muscular exercise, unless contra-indicated by cardiac disease; in such cases hot-air baths may be substituted. Henneberg, of Göttingen, states that, in cattle, rest and protection from cold favours the fattening process, and that converse conditions produce the opposite result. Bauer, of Munich, thinks it immaterial whether the fatty or the carbo-hydrate food be diminished, and suggests that the tastes of the patient be consulted. Unna agrees with Ebstein; as does also Leube, who, however, recommends a limited amount of alcohol, in order to cause active cell changes.

"NEW METHODS OF TREATING STOMACH DISEASES."—By Dr. Dujardin Beaumetz (*Therapeutic Gazette*, Dec., 1884). The author considers, more particularly, (1) Lavage, or washing out the stomach; and (2) Gavage, or forced feeding, especially by the use of meat powder. The apparatus he recommends is an ordinary soft œsophagus tube, and siphon action. Previous administration of potass. bromid. for two or three days, to diminish reflex irritation by the tube, is advised. One lavage a day, before breakfast, is usually sufficient. The fluid used should be lukewarm; and the process continued until the water returns clear, or nearly so. For simple lavage, the water should contain 60 grns. of sod. bicarb., or 90 grns. of sod. sulphate, to the quart. A 1. per cent. solution of boracic acid, and Belloc's charcoal powder, are indicated if fermentation occurs during digestion. When severe gastric pain exists, bismuthi subnit. (two tablespoonsful to a pint of water), aq. chloroform. (ʒii. to a quart), or solution of carbon bisulphide ($\frac{1}{3}$ saturated), are valuable. Lavage is recommended in dilatation of the stomach, from whatever cause; but not when ulceration is suspected or known to exist. Gavage, of nutritive mixtures, having meat powder as a basis, and milk, usually, as the vehicle, is especially useful in dyspepsia with anorexia, and in diarrhœa. A good powder is made as follows: Boil the meat for a short time; cut it into small pieces; dry over a water-bath, and grind in a coffee-mill. It is five times as strong as raw meat; is easily peptonised; and is free from the danger of conveying tænia.

PNEUMONIC ABSCESS.—In the *Journal of the American Med. Assoc.*, Dec. 19th, 1885, is an excellent paper on "Pneumonic Abscess," by Dr. E. F. Wells, which was read before the Chicago Medical Society. The conclusions arrived at from the consideration of a very large number of published cases and opinions (the references to which are given) are as follows: (1) The issue of pneumonic fever in abscess is rare, but this rarity has been generally overestimated. (2) The abscesses vary much in size, and are most frequently found at the base of the lung. (3) They may, in some cases, be caused by excessive jarring or other motion. (4) They are usually formed rapidly. (5) In some cases the purulent contents may degenerate into a cheesy mass, to again soften, liquefy, and be discharged. (6) The symptoms and signs are generally quite distinctive, and sufficient for an accurate diagnosis. (7) The majority of cases recover; but in a certain proportion a cure is impossible. (8) Expectant and medicinal treatment has, thus far, given the best results, and the majority of cases should be managed upon this plan; but under certain conditions the most radical measures for relief are not only justifiable, but imperatively demanded: thus, if the abscess be large and unopened, or if, although opened, it has an insufficient discharge, and the patient is steadily losing ground, and is evidently doomed to greatly prolonged illness, the good sense of the surgeon will decide that an operation is indicated. The necessary measures are, the formation of an external opening, by knife or cautery, and the maintenance, through this, of free drainage into an absorbent dressing, which must be frequently changed. The author objects to the use of antiseptic injections, especially if of a poisonous nature. His whole paper will well repay perusal.

HYOSCINE is a new drug which promises to be of some value. Dr. H. M. Wetherill, jun., gives, in the *Philadelphia Medical Times*, Dec. 26th, 1885, an account of what is already known of its action, and records his own experience of its use. It was discovered, and its properties first investigated, by Landenberg, of Germany. He was followed by Edelfsen, Gnauck, Emmert, &c., on the Continent; and, in America, by Dr. H. C. Wood and others. It is said to be prepared by the action of barium hydroxide on hyoscyamine. It is very volatile in the free state, and freely soluble in distilled water. The hydrobromate is the salt most used; the most reliable being that prepared by Merck, of Darmstadt. (A convenient formula is: Hyoscine hydrobrom., grn. i.; alcohol, ʒj.; aq. destill., ʒix. This contains $\frac{1}{60}$th grn. in ♏x.) On a healthy person, $\frac{1}{120}$th grn. produced, in 45 minutes, moderate mydriasis; dryness of throat, with hoarseness; suffusion of face and conjunctiva; a sense of fulness in the head, with vertigo; a rise in temperature of 1.5° F.; slowing of respiration and pulse (20 per cent.), the latter being also very full; general perspiration; muscular relaxation and impaired co-ordination; and a sense of mental wretchedness. He slept for nine hours, and on waking felt quite well; but the pupils were still a little dilated. This one case is typical of its usual effects. According to Dr. H. C. Wood (*Therapeutic Gazette*), "in mammals it acts as a spinal depressant, producing paralysis; a centric respiratory depressant, causing death by asphyxia; and, in enormous doses, a paralyser of the vaso-motor system." He states that, in medicinal doses, it causes no disturbance of secretions, or unpleasant results after its soporific effects have passed off. It was used, without relief, in facial neuralgia; but Prof. Edelfsen reports favourable results in enteralgia, pertussis, and asthma. Dr. Wetherill, after six months' use of it in the Pennsylvania Hospital for Insane, states that, as a hypnotic, it seldom fails, even in the daytime; and that toleration is not readily established —in this respect comparing favourably with hyoscyamine. The cases in which it was tried included acute delirious mania, chronic dementia, and the insomnia of agitated melancholia, of morphinomania, and of neurasthenia. It had a decided influence over the spasms in a case of tetanus, which, however, proved fatal from hyperpyrexia. He considers that it is the best general sedative at our disposal for calming the motor excitement of acute and chronic mental disorders, in their talkative, active, noisy, destructive, or violent phases. It is not always well borne; and in some cases has caused nausea, vomiting, anorexia, dysuria, and syncope, with a small, rapid, irregular pulse, and symptoms of partial paralysis of the pneumogastrics. Dr. H. C. Wood (*Therapeutic Gazette*, Sept., 1885) also reports two cases in which similar untoward symptoms occurred. He has found the drug valuable in spermatorrhœa and in erotomania. In the *Therapeutic Gazette*, p. 860, Dec., 1885, Mr. L. H. Taylor gives details of a case of puerperal mania, in which $\frac{1}{48}$th grn. caused several hours of quiet sleep. Dr. P. M. Wise, of the Willard Asylum for Insane (*New York Med. Rec.*, Jan. 30th, 1886), reports unfavourably as to its value as a sedative in active mania and melancholia, and also in pertussis; thus agreeing with Drs. Peterson and Langdon, in the same journal (Sept. 19th, 1885). He regards hyoscyamine as the more reliable drug.

Paraldehyde, if pure, is at 50° F. a solid crystalline substance, which melts at a rather higher temperature. The formula is $C_6\ H_{12}\ O_3$, and it is an isomer of acetic aldehyde, $C_2\ H_4\ O$. It is soluble in alcohol, and in 10 parts of water. The dose, as a hypnotic, is 30 to 60 grns., given at one time. (Paraldehyde f. ʒss., syr. aurantii f. ʒiv., pulv. tragacanth. co. gr. xx., aq. ad. ℥iii.) Compared with chloral, it has the following advantages: It is less irritating to the pharynx and stomach; is not a cardiac poison; and is a better antidote to strychnia. In nervous insomnias, especially when due to alcoholic abuse, and in maniacal conditions, it is much superior to chloral; but, like it, is of little use in insomnia dependent on pain. It has proved of service also in epilepsy, hysteria, and morphinomania. (Prof. Dujardin-Beaumetz, *Therapeutic Gazette*, Dec., 1885.)

Urethan, or Carbamic Ether ($C_2\,H_5\,O.\ NH_2.\ CO.$), has been lately recommended by Dr. von Jaksch, of Vienna (*Wiener Med. Blatter*, Nos. 33 and 34, 1885), as a valuable hypnotic. He had employed it 110 times in 20 cases of very different characters. The dose was usually .25 to 1 gram. (=3.85 to 15.4 grns.) It is well borne by the patients, and produces no unpleasant effects. The sleep lasts, as a rule, some hours. Merck, of Darmstadt, supplies it in the form of white crystals, readily soluble in water, odourless, and with a cooling saline taste. In the *Practitioner*, Feb., 1886, Dr. Saundby, of Birmingham, states that he has found it a very efficient hypnotic, in 2 grn. doses, in cases where paraldehyde had failed. The *latter*, too, has a decidedly unpleasant taste and smell, and causes the patient's breath to smell like a tippler's.

STROPHANTIN.—At the Cardiff meeting of the British Medical Association, 1885, Prof. Fraser, of Edinburgh, communicated an account of his researches on Strophantin, one of the many suggested substitutes for digitalis. It is a glucoside obtained from the seeds of a species of strophanthus, found widely in Central Africa, and used there as an arrow poison. He found that in frogs it has a direct influence on all striped muscle, causing powerful contraction; and that the heart was first affected, the beats becoming slow and more powerful. The blood pressure is raised; but not so much as by digitalis: the urine is increased, and the temperature lowered. In poisonous doses the heart is arrested in systole. He concludes that "strophanthus exerts a much more powerful action on the heart and less powerful action on the bloodvessels than digitalis." A great recommendation is its rapidity of action: this was well shown by its use in five patients suffering from mitral incompetence, with the usual sequelæ. In one case there was a marked improvement of the pulse within 20 minutes; and all the cases were much benefited. The dose is about $\frac{1}{50}$th grn.

CHOREA LARYNGIS.—Dr. G. W. Major records a case of this rare condition in the *Canada Med. and Surg. Journal*, Jan., 1886. He says: "A typical case of laryngeal chorea may be defined to be one in which there is an involuntary and uncontrollable cough during waking hours, absent during sleep, spasmodic in character, and accompanied by various sounds resembling the noise produced when straining, the barking of a dog, and so forth. There is associated with it spasm of the expiratory muscles. The speaking voice is normal; articulation is perfect; and the general health usually good." "On laryngoscopic examination, which is generally well borne, the act of coughing is found to be preceded by spasm of the laryngeal adductor muscles, bringing the vocal cords into close relation, often with an audible click, when the action of the expiratory muscles suddenly forces them apart, producing the somewhat characteristic cough. Between the acts of coughing the glottis assumes a variety of shapes, constantly changing its outline, indicating choreic movements of the intrinsic laryngeal muscles. The larynx itself presents but slight departure from a state of health, if we except trifling congestion of its mucous membrane, and occasionally, perhaps, a moderate degree of swelling." "In forming an opinion, it is essential that the hysterical element be carefully excluded." The case reported was that of a girl, aged 16, who had previously manifested general choreic symptoms. She steadily improved under arsenic, supplemented occasionally by zinci valerianas. Cocaine spray gave no relief, as might have been expected.

